# Identification of Novel circRNA-Based ceRNA Network Involved in the Pathogenesis of Gastric Cancer

**DOI:** 10.1155/2022/5281846

**Published:** 2022-05-31

**Authors:** Dengfa Peng, Li Feng, Huqing Li

**Affiliations:** First Surgical Department, The Central Hospital of Enshi Tujia and Miao Autonomous Prefecture, Hubei Province 445000, China

## Abstract

**Objective:**

Evidence increasingly shows that circular RNAs (circRNAs) are closely associated with tumorigenesis and cancer progression. However, the roles of circRNAs and the underlying mechanism behind these circRNAs in gastric cancer (GC) remain to be elucidated. This study is aimed at conferring a better understanding of GC pathogenesis with a specific focus on circRNA-based ceRNA action.

**Methods:**

circRNA expression profiles were downloaded from two Gene Expression Omnibus (GEO) microarray datasets, GSE152309 and GSE121445. Expression profiles of miRNAs and mRNAs were collected from The Cancer Genome Atlas (TCGA) database. The ceRNA network was constructed based on circRNA-miRNA pairs and miRNA-mRNA pairs. Functional and pathway enrichment analyses were performed to evaluate functional pathways of differentially expressed mRNAs (DEmRNAs). The PPI network was constructed by mapping DEmRNAs into the STRING database to identify hub genes, and then the DEcircRNA-DEmiRNA-hub gene subnetwork was constructed. The expression levels of candidate differentially expressed circRNAs (DEcircRNAs) in cancerous and matched noncancerous gastric tissues surgically resected from 52 GC patients were determined by the RT-qPCR analysis.

**Results:**

Differential expression analysis with Venn diagram analysis showed 11 overlapped DEcircRNAs (7 upregulated circRNAs and 4 downregulated ones) between cancerous tissues and noncancerous gastric tissues. The DEcircRNA-DEmiRNA-DEmRNA network was constructed, consisting of 2 DEcircRNAs, 7 DEmiRNAs, and 104 DEmRNAs. GO and KEGG pathway analyses revealed that 104 DEmRNAs might be associated with GC development and progression. The PPI network was constructed, yielding 16 hub genes, TFDP1, KRAS, LMNB1, MET, MYBL2, CDC25A, E2F5, HMGA1, HMGA2, CBFB, CBX3, CDC7, IGF2BP3, KIF11, PDGFB, and SMC1A, which were all upregulated in GC tissues compared with adjacent tumor-free gastric tissues. Based on the above hub genes in GC, the DEcircRNA-DEmiRNA-hub gene subnetwork was reconstructed based on hsa_circ_0000384 and hsa_circ_0000043, including 22 pairs of the upcircRNA-downmiRNA-upmRNA network. The expression levels of hsa_circ_0000384 and hsa_circ_0000043 were remarkably higher in GC tissues than those in matched adjacent tumor-free gastric tissues (*p* < 0.001), concurring with the bioinformatics results.

**Conclusion:**

Our study offers a better understanding of circRNA-related ceRNA regulatory mechanism in the pathogenesis of GC, highlighting two ceRNA networks based on hsa_circ_0000384 and hsa_circ_0000043.

## 1. Introduction

Gastric cancer (GC) is a common malignant tumor. Despite significant improvements in the diagnosis and prevention of GC, the incidence rate and mortality of GC remain a huge challenge worldwide. As reported in Global Cancer Statistics 2018, it was estimated that 1,033,701 people were newly diagnosed with GC, accounting for 5.7% of all confirmed cancer cases in 2018. The mortality of GC is the same as that of liver cancer. GC is responsible for 8.2% of cancer deaths in both sexes, second only to lung cancer (18.4%) and colorectal cancer (9.2%) [1]. The incidence of GC is closely associated with age. People aged over 50 are more likely to develop GC. The incidence rate is positively correlated with age and reaches a stable period between 55 and 80 years old [2]. In addition, other risk factors including environmental factors such as *Helicobacter pylori* infection and radiation, bad habits such as smoking and unhealthy diet such as salty food [3, 4], and genes such as CDH1 [5], PALB2, BRCA1, and RAD51C [6] have been identified for the occurrence of most GC. Most patients with GC usually have no obvious symptoms in the early stage and are diagnosed with advanced GC due to a low early diagnosis rate [7]. Therefore, there has been increasing interest in improving early detection technology. Actually, understanding the early molecular abnormalities of GC is of great significance for potential diagnosis and treatment. Circular RNA (circRNAs) is a kind of noncoding RNA molecule that has a closed-ring structure formed by a covalent bond, without 5′ end cap and 3′ end poly (a) tail. circRNAs are produced from nonsequential back-splicing of precursor mRNA (pre-mRNA) in highly diverged eukaryotes and are less affected by RNA exonuclease, signifying its expression being more stable and difficult to degrade. A considerable group of circRNAs is composed of exon sequences, which are conservative in different species and specifically expressed in tissues and different development stages [8]. circRNAs perform several biological functions by acting as a transcription regulator, microRNA (miRNA) sponge, and protein template [9, 10]. Accordingly, the competitive endogenous RNA (ceRNA) hypothesis has emerged that circRNAs shared microRNA (miRNA) binding sites and compete for posttranscriptional control, which is becoming a new paradigm of ncRNA regulation [11]. circRNAs are ideal candidates for diagnostic biomarkers and therapeutic interventions in various diseases due to their stability, conservation, and high abundance in body fluids, and their diagnostic value has been well established in osteoarthritis [12], cardiovascular disease [13], neurological disorders [14], and breast cancer [15]. Studies have confirmed that circRNAs are involved in the proliferation, invasion, metastasis, and apoptosis of GC [16, 17]. However, investigations on novel circRNAs as potential diagnostic biomarkers and related regulator networks in the development of GC remain limited.

In the study, we first performed differential analysis using the circRNA profiles of GSE152309 and GSE121445 both deposited in the Gene Expression Omnibus (GEO) (http://www.ncbi.nlm.nih.gov/gds/) and miRNA and mRNA profiles from The Cancer Genome Atlas (TCGA) database, in a bid to acquire differentially expressed circRNAs, miRNAs, and mRNAs (abbreviated as DEcircRNAs, DEmiRNAs, and DEmRNAs) between cancerous tissues and noncancerous gastric tissues. In the next step, the DEcircRNA-DEmiRNAs-DEmRNAs network was constructed, and the protein-protein interaction (PPI) network was established to obtain the hub gene, on the basis of which the circRNA-miRNA-hub gene subnetwork regulation module was acquired. Subsequent clinical validation was carried out on cancerous and tumor-free gastric tissues surgically resected from GC patients. This study is aimed at conferring a better understanding of GC pathogenesis with a specific focus on circRNA-based ceRNA action.

## 2. Methods

### 2.1. Acquisition of Raw Data

Two circRNA profiles were obtained from GSE152309 and GSE121445, both deposited in the GEO. The GSE152309 was generated on the GPL18573 platform, including 5 paired tumor and adjacent nontumor tissues of GC patients. The GSE121445 was generated on the GPL20795 platform, containing 3 paired cancerous tissues and adjacent noncancerous gastric tissues. GSE152309 and GSE121445 are selected for identification of DEcircRNAs between cancerous tissues and noncancerous gastric tissues considering the following: (i) sample type: gene expression profiles of these two datasets are both sourced from cancer tissue samples; ii) profiling by high-throughput sequencing; and iii) supplements with clear series matrix files and gene symbols. The miRNA and mRNA profiles of stomach adenocarcinoma patients were obtained from TCGA database, including 436 GC and 41 adjacent noncancerous gastric tissue samples and 375 GC and 32 adjacent noncancerous gastric tissue samples, respectively. Raw data were normalized and log2-transformed. The limma package loaded in the R/Bioconductor software was employed to analyze DEcircRNAs, DEmiRNAs, and DEmRNAs using (|log (fold change [*FC*])| > 1.5 and adjusted *p* < 0.05 as cutoff values, between cancerous tissues and noncancerous gastric tissues.

### 2.2. Prediction of miRNA Binding Sites and miRNA Target Genes

Common DEcircRNAs between GSE152309 and GSE121445 datasets were mapped into the starBase to obtain DEcircRNA-miRNA pairs. DEmRNAs were mapped into miRMap, miRanda, miRDB, TargetScan, and miTarBase, five miRNA-mRNA target prediction databases, to obtain miRNA-DEmRNA pairs.

### 2.3. Construction of the circRNA-miRNA-mRNA ceRNA Network

The DEcircRNA-miRNA-DEmRNA regulatory network was constructed using a combination of DEcircRNA-miRNA pairs and miRNA-DEmRNA pairs based on the principle of the ceRNA network. To construct a GC-related ceRNA network, the DEcircRNA-DEmiRNA-DEmRNA and DEcircRNA-DEmiRNA-hub gene subnetworks were constructed and visualized using the Cytoscape 3.4.0 software.

### 2.4. Gene Ontology and Pathway Enrichment Analysis

Gene ontology (GO) terms and KEGG pathways are important indicators to distinguish essential genes from nonessential genes. GO terms typically describe three related functions including biological process (BP), cellular component (CC), and molecular function (MF). The “clusterProfiler” software package in the R/Bioconductor was used to determine the basic biochemical metabolism and signal transduction pathways related to the target gene by *p* value < 0.05 as the cutoff value. The KEGG pathway database displays graphical maps of biochemical pathways obtaining metabolic and regulatory pathways, and it is applied to systematically analyze gene networks and molecular networks. The KEGG enrichment analysis was performed based on hypergeometric distribution by Fisher's test through the use of the “clusterProfiler” package in R/Bioconductor. Calculation (*p* value) of each pathway was carried out and the screening criteria were defined as *p* value < 0.05.

### 2.5. Screening of Hub Genes by PPI Network

The Search Tool for the Retrieval of Interacting Genes (STRING online tool) was employed to screen hub genes. If the interactions with a medium confidence > 0.4, it means the interaction of hub genes is significant. The Cytoscape plugin cytoHubba was employed to construct the integrated regulatory networks.

### 2.6. Human Tissue Samples

We collected 52 pairs of cancerous and adjacent tumor-free gastric tissues surgically resected from GC patients who were admitted into our hospital from January 2020 to January 2021. Patients included should be histologically or cytologically diagnosed as primary GC, cancer staging performed according to the 7th edition of the American Joint Committee on Cancer (AJCC) [18], and they were excluded from the study if they had been diagnosed with other malignancies. No patients had received radiotherapy or chemotherapy before tissue specimen collection. Matched adjacent tumor-free gastric tissues had a 5-10 cm distance from tumors, in which the absence of tumor cells was histologically confirmed. All included GC patients, including 41 males and 11 females, signed an informed consent form, and human tissue specimen collection was approved by the Ethics Committee of the Central Hospital of Enshi Tujia and Miao Autonomous Prefecture.

### 2.7. Real-Time Quantitative Polymerase Chain Reaction (RT-qPCR) Validation

The total RNA was isolated from cancerous and tumor-free gastric tissues using TRIzol reagents (Invitrogen, USA), and then the PrimeScript RT kit (Takara, Dalian, China) was applied for the generation of cDNA. The SYBR-Green PCR kit (Roche Diagnostics, USA) was carried out to RT-qPCR through a StepOnePlus Real-Time PCR system (Applied Biosystems, USA). The sequence information of the primers used for the qPCR was as follows: hsa_circ_0000384, 5′-TGTTTCATCAAGACCATCTGTTC-3′ (sense) and 5′-CCTAGGAGGTAGTGCCTTTCTTC-3′ (antisense); hsa_circ_0000043, 5′-GCATGGAGCCTCTTCAGTTTG-3′ (sense) and 5′-CCATTGGTCTTGTCCATCAC-3′ (antisense). The results were analyzed using the 2^-*ΔΔ*Ct^ method and normalized to GAPDH, 5′-ATGGAGAAGGCTGGGGCTC-3′ (sense) and 5′-AAGTTGTCATGGATGACCTTG-3′ (antisense).

### 2.8. Statistical Analysis

Relative expression levels of hsa_circ_0000384 and hsa_circ_0000043 between cancerous and matched noncancerous gastric tissues were compared using the paired *t*-test. Statistical analysis and figure creation were performed using GraphPad Prism Version 8. A possibility of *p* < 0.05 reflects a significant difference.

## 3. Results

### 3.1. Identification of DEcircRNAs in GC

**Figure 1:**
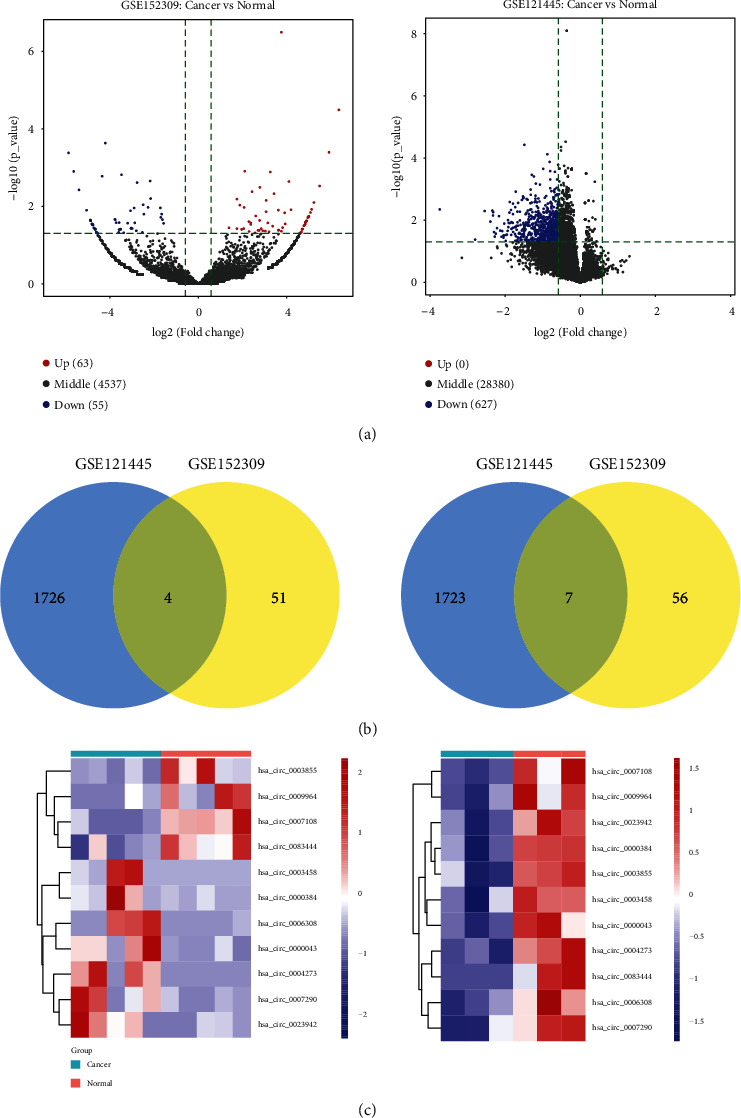
Identification of DEcircRNAs between GC and adjacent tumor-free gastric tissue samples by differentially analyzing the raw data of the GSE152309 (5 paired tumor and nontumor tissues) and GSE121445 (3 paired tumor and nontumor tissues) datasets. (a) The volcano plots. (b) Venn diagram showing 11 overlapped DEcircRNAs in two datasets. (c) The heatmaps showing expression diversity of 11 overlapped DEcircRNAs in two datasets, among which color more red indicates higher expression and more blue indicates lower expression.

We differentially analyzed the raw data of the circRNA profile of GC patients in the two microarray datasets, GSE152309 (last update date: Aug 27, 2021) and GSE121445 (last update date: Mar 23, 2020), and, respectively, identified 118 DEcircRNAs (63 upregulated circRNAs and 55 downregulated ones) and 627 DEcircRNAs (only downregulated circRNAs) between cancerous tissues and noncancerous gastric tissues (Figure 1(a)). For identifying the common DEcircRNAs in both two microarray datasets, we used |logFC| > 0 and adjusted *p* < 0.05 as cutoff values to perform a differentially expression analysis of circRNAs in the GSE121445 database. Further Venn diagram analysis showed 7 overlapped upregulated circRNAs and 4 overlapped downregulated ones (Figures 1(b) and 1(c)).  Table 1 lists the basic characteristics of 11 DEcircRNAs.

### 3.2. Identification of DEmiRNAs and DEmRNAs in GC

**Figure 2:**
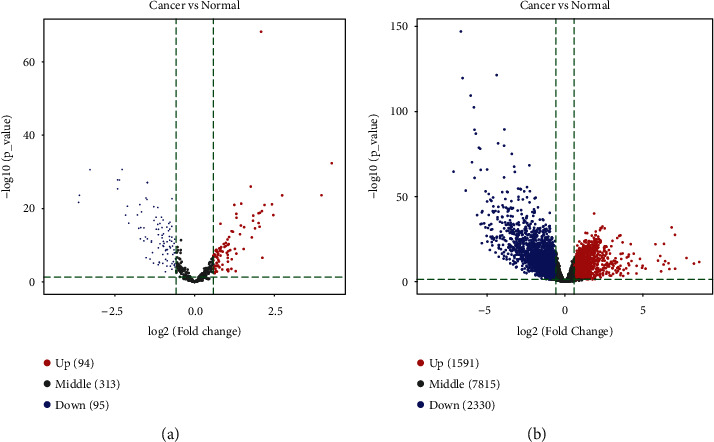
Identification of DEmiRNAs (a) between GC tissue (*n* = 436) and adjacent tumor-free gastric tissue (*n* = 41) samples and DEmRNAs (b) between GC tissue (*n* = 375) and adjacent tumor-free gastric tissue (*n* = 32) samples by differentially analyzing the raw data deposited in TCGA-STAD.

We next differentially analyzed the miRNA and mRNA profiles of stomach adenocarcinoma patients deposited in TCGA-STAD and found 189 DEmiRNAs (|log2(*RPM*)| > 1.5 and adjusted *p* < 0.05) between GC tissue (*n* = 436) and adjacent tumor-free gastric tissue (*n* = 41) samples, including 94 upregulated miRNAs and 95 downregulated ones (Figure 2(a)), 3921 DEmRNAs (|*logFC*| > 1.5 and adjusted *p* < 0.05) between GC tissue (*n* = 375) and adjacent tumor-free gastric tissue (*n* = 32) samples, including 1591 upregulated mRNAs and 2330 downregulated ones (Figure 2(b)).

### 3.3. Construction of DEcircRNA-DEmiRNA-DEmRNA Regulatory Network in GC

**Figure 3:**
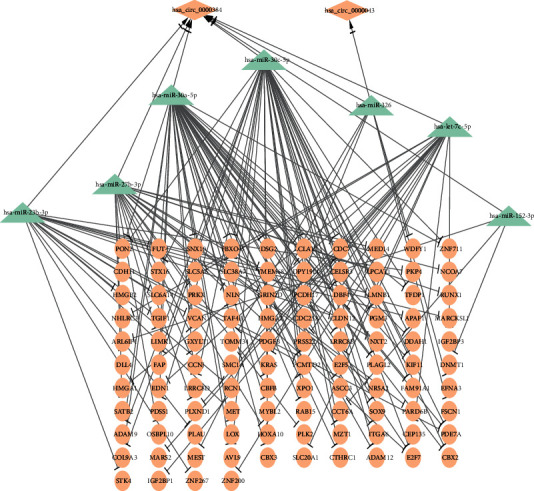
The DEcircRNA-DEmiRNA-DEmRNA network in GC, consisting of 2 DEcircRNAs, 7 DEmiRNAs, and 104 DEmRNAs.

To better study the interaction of circRNAs and miRNAs in the ceRNA network of GC, we mapped 11 DEcircRNAs into the starBase and identified 70 pairs of interacting DEcircRNAs and miRNAs. We then predicted miRNAs targeting 3921 DEmRNAs among five databases (miRMap, miRanda, miRDB, TargetScan, and miTarBase) and identified 20320 pairs of interacting miRNAs and DEmRNAs (11870 pairs based on downregulated DEmRNAs and 8450 pairs based on upregulated DEmRNAs). As indicated by the ceRNA hypothesis, circRNAs share miRNA binding sites and compete for posttranscriptional control, referring to two paradigms of regulation: downregulated circRNAs lead to upregulated miRNAs and thus contribute to downregulated mRNA, and upregulated circRNAs lead to downregulated miRNAs and thus contribute to upregulated mRNA. We abbreviated these two paradigms of ceRNA regulation as the downcircRNA-upmiRNA-downmRNA network and upcircRNA-downmiRNA-upmRNA network. Therefore, we intersected 70 pairs of interacting DEcircRNA-miRNA and 20320 pairs of interacting miRNA-DEmRNA to initially construct the DEcircRNA-miRNA-DEmRNA network of GC. The miRNAs in the DEcircRNA-miRNA-DEmRNA network were crosschecked against 189 DEmiRNAs retrieved from TCGA database, and the DEcircRNA-DEmiRNA-DEmRNA network was finally constructed, consisting of 2 DEcircRNAs, 7 DEmiRNAs, and 104 DEmRNAs (Figure 3).

### 3.4. GO Annotation and KEGG Pathway Analyses of DEmRNAs in GC

**Figure 4:**
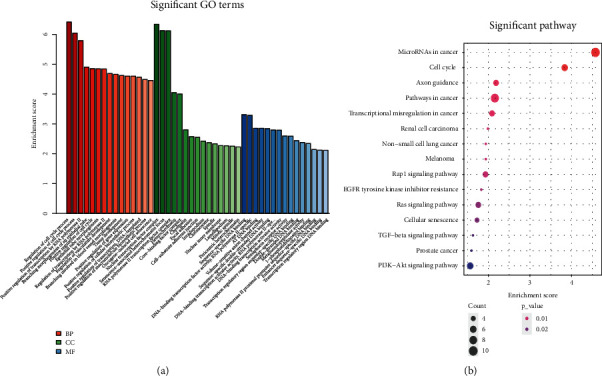
The DEmRNAs in the DEcircRNA-DEmiRNA-DEmRNA network were analyzed by GO annotation and KEGG pathway. (a) The top 15 GO terms enriched by DEmRNAs based on BP, CC, and MF categories. (b) Pathways significantly enriched by the DEmRNAs in the KEGG database. Red indicates small *p* value, and blue indicates large *p* value; the size of the bubbles indicates the degree of enrichment, and larger bubbles reflect larger gene ratio.

To evaluate the main functional pathways of GC, we conducted GO annotation and KEGG pathway analyses of 104 DEmRNAs in GC. GO analysis revealed that the 104 DEmRNAs were enriched in 420 GO terms (*p* < 0.05).  Figure 4(a) shows the top 15 GO biological processes significantly enriched by DEmRNAs based on BP, CC, and MF categories, including regulation of cell cycle process (GO: 0010564), positive regulation of transcription by RNA polymerase II (GO: 0045944), nuclear transcription factor complex (GO: 0044798), and senescence-associated heterochromatin focus (GO: 0035985).  Figure 4(b) shows the top 15 KEGG pathways significantly enriched by DEmRNAs (*p* < 0.05) by DEmRNAs including microRNAs in cancer (hsa05206), cell cycle (hsa04110), and axon guidance (hsa04360).

### 3.5. Construction of DEcircRNA-DEmiRNA-Hub Gene Subnetwork

**Figure 5:**
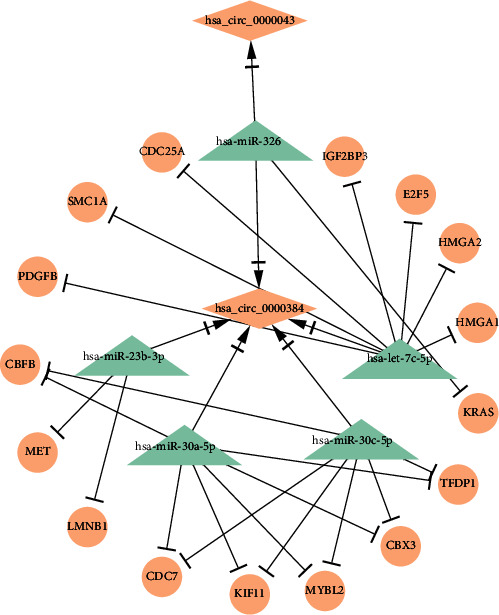
Construction of DEcircRNA-DEmiRNA-hub gene subnetwork.

**Figure 6:**
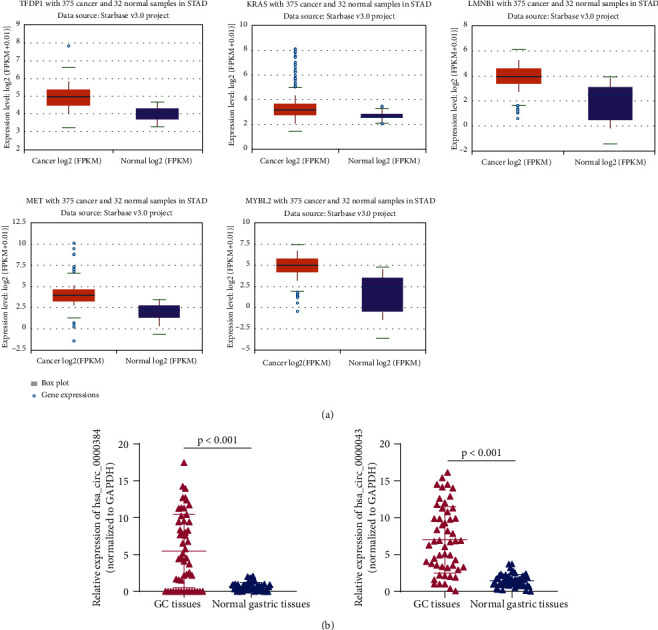
(a) Expression patterns of TFDP1, KRAS, LMNB1, MET, and MYBL2 in GC using the starBase database. (b) Relative expression levels of hsa_circ_0000384 and hsa_circ_0000043 in cancerous and matched noncancerous gastric tissues surgically resected from 52 GC patients were determined by RT-qPCR, normalized to GAPDH.

The PPI network was constructed by mapping 104 DEmRNAs between GC and adjacent tumor-free gastric tissue samples into the STRING database, including 16 nodes (Table 2). These 16 hub genes, TFDP1, KRAS, LMNB1, MET, MYBL2, CDC25A, E2F5, HMGA1, HMGA2, CBFB, CBX3, CDC7, IGF2BP3, KIF11, PDGFB, and SMC1A were all upregulated in GC tissues compared with adjacent tumor-free gastric tissues. Based on the above hub genes in GC, the DEcircRNA-DEmiRNA-hub gene subnetwork was reconstructed, including 22 pairs of the upcircRNA-downmiRNA-upmRNA network, as shown in Figure 5 and Table 3. In addition, expression patterns of the top 5 hub genes, TFDP1, KRAS, LMNB1, MET, and MYBL2, were also investigated using the starBase database. It was found these 5 hub genes exhibited high expression levels in GC tissue samples compared with adjacent tumor-free gastric tissues (*p* < 0.05, Figure 6(a)).

### 3.6. RT-qPCR Validation

To check the credibility of the bioinformatics results, we determine the expression levels of hsa_circ_0000384 (also known as circMRPS35) and hsa_circ_0000043 (also known as circPUM1) in cancerous and matched noncancerous gastric tissues surgically resected from 52 GC patients by RT-qPCR analysis. Results were expressed as mean ± standard deviation and analyzed by using the paired *t*-test. The expression levels of hsa_circ_0000384 and hsa_circ_0000043 were remarkably higher in GC tissues than those in matched adjacent tumor-free gastric tissues (*p* < 0.001, Figure 6(b)). The RT-qPCR results from 52 GC patients were consistent with the bioinformatics results.

## 4. Discussion

The first case of gastric cancer (GC) was documented in the Ebers Papyrus which can be traced back to 1600 BC, and Galen discussed its etiology of attacking the body through the skin in 100 AD afterwards [19]. In the clinical field, GC is histologically divided into two main types consisting of diffuse subtype and intestinal subtype based on Lauren's classification system [20]. The World Health Organization (WHO) classification of digestive system tumors 5th Edition classifies GC into five histologic subtypes [21]. GC, as a common malignant tumor, is the third leading cause of cancer-related death. At present, there is no simple and effective screening method for early detection of GC, resulting in an increase in the number of advanced patients. With the significant improvement in molecular biology technology, abnormal expression of circRNA in tumor development, such as hepatocellular carcinoma [22], lung adenocarcinoma [23], and GC [24], has been confirmed. However, limited research on ceRNA regulatory network in GC was observed. Here, we revealed the circRNA-mediated ceRNA regulatory network in GC on the basis of the comprehensive analysis of GEO and TCGA databases.

In recent years, it has been found that a large number of circRNAs are involved in the occurrence and development of solid tumors including GC [25, 26]. The emerging evidence suggests that circRNA may become an important new biomarker and target for the diagnosis and prognosis of GC [27]. For example, Zhang et al. indicated that overexpression of has_circ_0032627 was associated with poor survival in patients with GC, and has_circ_0032627 knockdown contributed to the inhibition of GC metastasis via inactivating the NRAS/MEK1/ERK1/2 signaling pathway and regulating the miR-502-5p expression [28]. circLMTK2 acts as a miR-150-5p sponge and facilitates proliferation and metastasis in GC [29]. circNHSL1 acts as a sponge to positively regulate SIX1, the target of miR-1306-3p [30]. circ-RanGAP1 sponges miR-877-3p upregulate the VEGFA expression, thus promoting cancer cell aggressiveness in GC [31]. The present study constructed the DEcircRNA-DEmiRNA-DEmRNA network consisting of 2 DEcircRNAs, 7 DEmiRNAs, and 104 DEmRNAs. The RT-qPCR analysis confirmed that hsa_circ_0000384 (also known as circMRPS35) and hsa_circ_0000043 (also known as circPUM1) levels elevated in GC tissues compared with adjacent tumor-free gastric tissues. Upregulated expression of hsa_circ_0000384 and hsa_circ_0000043 accelerated GC cell proliferation and invasion. Another study reported by Li et al. [32] revealed that increased expression of hsa_circ_0000384 was found in patients with hepatocellular carcinoma. Knockdown of hsa_circ_0000384 expression inhibited the proliferation and migration of tumor cells. Elevated levels of hsa_circ_0000043 are closely related to the occurrence of lung adenocarcinoma. Impairment of tumor cells' proliferation, migration, and invasiveness is associated with the silencing of hsa_circ_0000043 expression. Furthermore, hsa_circ_0000043 promotes Cyclin D1 and Bcl-2 protein expression by sponging miR-326 [9].

circRNAs can act as a miRNA sponge and competitively bind to miRNAs to regulate their target mRNA. Its function concerning the inhibition or promotion of GC progression has been clarified [33, 34]. The present study also constructed the DEcircRNA-DEmiRNA-hub gene subnetwork including 22 pairs of the upcircRNA-downmiRNA-upmRNA network, and it was found that 5 hub genes consisting of TFDP1, KRAS, LMNB1, MET, and MYBL2 revealed a high expression level in GC tissue samples compared with adjacent tumor-free gastric tissue samples. The hsa_circ_0000384 mediated TFDP1 expression by sponging has-miR-30a-5p and has-miR-30c-5p. Morimoto et al. [35] demonstrated that TFDP1 was identified as a target gene of miR-4711-5p in colon cancer cells. miR-4711-5p significantly inhibited tumor growth by downregulating TFDP1 mRNA and protein levels. Yasui et al. also showed TFDP1 gene was overexpressed in some cell lines of hepatocellular carcinomas [36]. In this study, hsa_circ_0000043 and hsa_circ_0000384 positively regulate KRAS gene expression by mediating has-miR-326. RAS is the most frequently mutated oncogene in cancer, and it includes KRAS, HRAS, and NRAS subtypes of which KRAS is commonly seen in cancer [37]. KRAS gene mutations occurred in approximately 35-45% of colorectal cancer patients [38]. The present study also indicated that hsa_circ_0000384 accelerated GC cell proliferation by upregulating MET and LMNB1. Downregulation of LMNB1 contributed to alleviating cellular defects in DYT1 MNs, resulting in improvement in hereditary neuromotor disorder [39]. Patients with non-small-cell lung cancer suffered from MET gene mutations, and treatment of capmatinib inhibited tumor activity in the patients with MET mutations [40]. MYBL2 is essential to regulate cell proliferation, differentiation, and DNA repair, and its overexpression is associated with shorter overall survival in breast cancer patients. It can be used as a biomarker in breast cancer progression [41]. This result was similar to ours showing increased MYBL2 levels that promoted GC development. However, since the results are based on the database, further functional studies are required to verify the involvement of these pairs of circRNA-miRNA-mRNA in GC.

We must acknowledge the presence of shortcomings when interpreting our results. First, although two circRNA profiles were detected by high-throughput sequencing of human cancerous or noncancerous gastric tissues, DEcircRNAs between cancerous or noncancerous gastric tissues were more balanced in up- and downregulation in the GSE152309 than the GSE121445, which may be indicative of the heterogeneity of raw data. Second, the sample size of clinical human tissues for RT-qPCR validation of circRNAs of interest was relatively small, which may weaken the reliability of clinical data. Third, given the preliminary nature of our study, further functional studies were required to elucidate GC pathogenesis focusing on circRNA-based ceRNA action.

In conclusion, we performed bioinformatics analysis and clinical validation which provided circRNA-mediated ceRNA networks involved in the progression of GC in order to understand the pathogenesis of the disease. The study revealed that hsa_circ_0000384 and hsa_circ_0000043 were upregulated in the context of GC, sponged antioncogenic miRNA, and posttranscriptionally regulated gene expression in GC, which might be served as promising biomarkers for GC diagnosis and targeted therapy.

## Figures and Tables

**Table 1:** The basic characteristics of 11 DEcircRNAs between cancerous tissues and noncancerous gastric tissues.

circRNA ID	Position	Genomic length	Strand	Best transcript	Gene symbol	Regulation
hsa_circ_0003458	chr9:115013208-115060196	46988	—	NM_001163790	PTBP3	Up
hsa_circ_0004273	chr17:60111147-60112969	1822	—	NM_005121	MED13	Up
hsa_circ_0006308	chr5:38991050-39021238	30188	—	NM_152756	RICTOR	Up
hsa_circ_0007290	chrX:44383247-44386611	3364	—	NM_173794	FUNDC1	Up
hsa_circ_0023942	chr11:85733409-85742653	9244	—	NM_007166	PICALM	Up
hsa_circ_0000043	chr1:31465236-31468067	2831	—	NM_014676	PUM1	Up
hsa_circ_0000384	chr12:27867712-27877119	9407	+	NM_001190864	MRPS35	Up
hsa_circ_0007108	chrX:24190831-24197887	7056	+	NM_001178085	ZFX	Down
hsa_circ_0003855	chr12:12672795-12674397	1602	—	NM_030640	DUSP16	Down
hsa_circ_0009964	chr1:12335881-12338095	2214	+	NM_015378	VPS13D	Down
hsa_circ_0083444	chr8:17601112-17613470	12358	—	NM_001001924	MTUS1	Down

**Table 2:** The hub genes in the PPI network and their interaction degree.

String node	Gene name	String ID	Freq
TFDP1	TFDP1	ENSP00000364519	6
KRAS	KRAS	ENSP00000256078	4
LMNB1	LMNB1	ENSP00000261366	4
MET	MET	ENSP00000317272	4
MYBL2	MYBL2	ENSP00000217026	4
CDC25A	CDC25A	ENSP00000303706	3
E2F5	E2F5	ENSP00000398124	3
HMGA1	HMGA1	ENSP00000399888	3
HMGA2	HMGA2	ENSP00000437621	3
CBFB	CBFB	ENSP00000415151	2
CBX3	CBX3	ENSP00000336687	2
CDC7	CDC7	ENSP00000393139	2
IGF2BP3	IGF2BP3	ENSP00000258729	2
KIF11	KIF11	ENSP00000260731	2
PDGFB	PDGFB	ENSP00000330382	2
SMC1A	SMC1A	ENSP00000323421	2

**Table 3:** The DEcircRNA-DEmiRNA-hub gene subnetwork.

DEcircRNA	DEmiRNA	DEmRNA
hsa_circ_0000043	has-miR-326	KRAS
hsa_circ_0000384	has-miR-326	KRAS
hsa_circ_0000384	has-miR-23b-3p	MET
hsa_circ_0000384	has-miR-23b-3p	LMNB1
hsa_circ_0000384	has-miR-30a-5p	CBFB
hsa_circ_0000384	has-miR-30a-5p	CDC7
hsa_circ_0000384	has-miR-30a-5p	KIF11
hsa_circ_0000384	has-miR-30a-5p	MYBL2
hsa_circ_0000384	has-miR-30a-5p	CBX3
hsa_circ_0000384	has-miR-30a-5p	TFDP1
hsa_circ_0000384	has-miR-30c-5p	CBFB
hsa_circ_0000384	has-miR-30c-5p	CDC7
hsa_circ_0000384	has-miR-30c-5p	KIF11
hsa_circ_0000384	has-miR-30c-5p	MYBL2
hsa_circ_0000384	has-miR-30c-5p	CBX3
hsa_circ_0000384	has-miR-30c-5p	TFDP1
hsa_circ_0000384	has-let-7c-5p	HMGA1
hsa_circ_0000384	has-let-7c-5p	HMGA2
hsa_circ_0000384	has-let-7c-5p	E2F5
hsa_circ_0000384	has-let-7c-5p	IGF2BP3
hsa_circ_0000384	has-let-7c-5p	CDC25A
hsa_circ_0000384	has-let-7c-5p	SMC1A
hsa_circ_0000384	has-let-7c-5p	PDGFB

## Data Availability

The GSE152309 and GSE121445 microarray datasets and miRNA and mRNA profiles supporting the results displayed in the study could be downloaded from the GEO database (https://www.ncbi.nlm.nih.gov/gds) and TCGA database (https://tcga-data.nci.nih.gov/), respectively.
